# Enhancing cross-cultural applicability in recovery colleges: A global Delphi study protocol

**DOI:** 10.1371/journal.pone.0332729

**Published:** 2025-09-30

**Authors:** Yasuhiro Kotera, Tesnime Jebara, Vanessa Lawrence, Simran Takhi, Amy Ronaldson, Simon Lawrence, Vanessa Kellermann, Agnieszka Kapka, Peter Bates, Claire Henderson, Mike Slade

**Affiliations:** 1 School of Health Sciences, Institute of Mental Health, University of Nottingham, Nottingham, United Kingdom; 2 Center for Infectious Disease Education and Research, The University of Osaka, Osaka, Suita, Japan; 3 Department of Social Sciences, Azerbaijan University, Baku, Azerbaijan; 4 Health Service and Population Research Department, King’s College London, Institute of Psychiatry, Psychology and Neuroscience, London, United Kingdom; 5 RECOLLECT Lived Experience Advisory Panel, London, United Kingdom; 6 Nord University, Faculty of Nursing and Health Sciences, Health and Community Participation Division, Namsos, Norway; University of Kansas School of Medicine Wichita, UNITED STATES OF AMERICA

## Abstract

**Background:**

Recovery Colleges (RCs) offer an innovative model of mental health support that blends co-production with adult learning to promote personal recovery and social inclusion. While evidence supports their effectiveness, most RC research and practice have been developed in Western contexts, raising concerns about cross-cultural applicability. The RECOLLECT Change Model (RCM) and RECOLLECT Fidelity Measure (RFM) were developed in England to characterise RC mechanisms and assess fidelity. Our previous studies have identified cultural influences on the RC operational model, however how to address these influences remains unknown. Given the increasing global interest in RCs, the aims of this study are to (a) identify the level of cultural influence on the RCM mechanisms and RFM items, and (b) provide recommendations to inform cross-cultural applicability of RCM and RFM.

**Methods:**

This global Delphi study follows Belton’s six-step methodology and uses a decentring approach to cross-cultural research that seeks to extend the relevance of tools developed in a single culture to multiple cultural contexts. Experts will be recruited via the RECOLLECT International Research Consortium, covering 31 countries across six continents. We aim to recruit approximately 100 panellists with at least three years’ RC experience. Data collection will occur via Microsoft Forms across iterative Delphi rounds. Panellists will rate the importance and cultural difficulty of RCM and RFM items, provide feedback on culturally aligned response types, and suggest revisions for improved cultural fit. Quantitative data will be analysed using non-parametric statistics and a collapsed three-point Likert scale to address cross-cultural response bias. Qualitative responses will be analysed using descriptive content analysis informed by Hofstede’s cultural dimension theory. Member checking will be conducted after the final round to enhance trustworthiness.

**Discussion:**

This study will identify which RCM and RFM components are cross-culturally applicable and which require adjustment, contributing to the balance between fidelity and fit in mental health approaches. By developing culturally informed recommendations, this study aims to expand the accessibility and relevance of RC frameworks across diverse settings. Findings will benefit RC practitioners, researchers, and policymakers seeking to improve service delivery and recovery outcomes in culturally meaningful ways.

## Introduction

Recovery Colleges (RCs) are an innovative approach to mental health support, combining co-production and adult learning principles to promote personal recovery and social inclusion [1]. Established in England in 2009, RCs have expanded to 28 countries, with 221 RCs globally as of 2022 [2]. RCs provide inclusive educational environments where people with mental health problems, carers, and staff collaboratively access education, develop skills, and build support networks. [3]. Through self-directed learning, RCs empower students to lead fulfilling lives, enhance social roles, and improve overall well-being [4].

The effectiveness and cost-effectiveness of RCs are supported by emerging evidence, including improved self-esteem, quality of life, and reduced internalised stigma among students, [5] alongside increased motivation and skill development for staff [6]. Preliminary studies highlight cost savings, such as reduced healthcare utilisation [7]. However, standardised operational guidance for RCs remains limited, which is being addressed in the Recovery Colleges Characterisation and Testing (RECOLLECT) 2 programme [8]. The RECOLLECT Change Model (RCM) characterises the mechanisms of action within RCs, [9] and the RECOLLECT Fidelity Measure (RFM) assesses RC fidelity to best practice [10]. These tools were developed through co-production with RC students, staff, and researchers, and were informed by evidence synthesis and stakeholder interviews to ensure they reflect both best practice and lived experience [9,10].

The RCM identifies mechanisms underlying RC operations, comprising empowering environment, shifting the balance of power, enabling different relationships, and facilitating personal growth [9]. These mechanisms produce outcomes including self-confidence, self-management, and social engagement [9]. The RFM evaluates RC adherence to these core principles through a 12-item fidelity tool, encompassing modifiable and non-modifiable components. Non-modifiable component items are responded on a three-point ordinal scale from 0 (low fidelity) to 2. The five modifiable component items are assessed using a categorical variable with either Type 1 or Type 2 responses, which are pre-defined for each item to reflect two distinct but valid ways RCs may may operate or be structured. The modifiable components can only inform the types instead of high-low fidelity [10]. The measure satisfies scaling assumptions, demonstrating adequate internal consistency (0.72), test–retest reliability (0.60), content validity and discriminant validity. Table 1 presents a summarised version of RFM [10].

**Table 1:** Summarised version of the RECOLLECT Fidelity Measure.

Non-modifiable Components		0 (low fidelity) to 2
(1) Equality		0–2
(2) Adult Learning		0–2
(3) Tailoring to the Student		0–2
(4) Co-production		0–2
(5) Social Connectedness		0–2
(6) Community Focus		0–2
(7) Commitment to Recovery		0–2
*Total Fidelity Score (Sum of 1–7)*		*0–14*
**Modifiable Components**	**Type 1**	**Type 2**
(8) Available to All	The Recovery College is available to all.	The Recovery College is limited to specific groups.
(9) Location	The Recovery College is based in a community location that is not shared with health, social care or other statutory services.	The Recovery College is based in a location which is shared with health, social care or other statutory services.
(10) Distinctiveness of Course Content	Any topic can be offered as a course, irrespective of whether it is available in mainstream adult education settings.	Only topics not available in mainstream adult education settings are offered.
(11) Strengths-based	A focus on strengths (not problems) is implicit in the college.	A focus on strengths (not problems) is explicit in the college, in addition to dimensions 1–7 above.
(12) Progressive	There is a focus on ‘being’ and ‘belonging’, not on goal-setting.	There is a focus on ‘becoming’ and a strong emphasis on goal-setting and change.

Despite their global growth, our previous studies have identified that RC research has been primarily concentrated in Western, Educated, Industrialised, Rich, and Democratic (WEIRD) countries, raising questions about their cross-cultural applicability [2,11–14]. All published reviews on RCs [5,6,15–18] have identified studies conducted in WEIRD contexts only, limiting understanding of broader cultural influences. Evidence from other mental health interventions shows that culturally adapted treatments yield significantly better outcomes, [19] underscoring the importance of considering cultural influences. However, while cultural adaptation focuses on tailoring interventions to fit a specific cultural context, this study aims to examine the broader cross-cultural applicability of RCs—that is, their potential to be relevant and useful across diverse cultural settings [20].

In this study, we conceptualise culture as “the collective programming of the mind that distinguishes the members of one group or category of people from others,” drawing on Hofstede’s widely used cross-cultural framework [21]. Rather than grouping countries by broad regions (e.g., Europe vs Asia) or historical categories (e.g., Commonwealth vs non-Commonwealth), we will use Hofstede’s cultural dimensions to identify patterns of cultural influence. This allows for a more granular understanding of cultural differences. For example, while Japan is often seen as collectivistic in Western comparisons, Hofstede’s metrics reveal that Japan is more individualistic than many other Asian countries, highlighting the value of this approach for nuanced cross-cultural considerations.

## Materials and methods

The PLOS ONE Study Protocol Template [22] was used to guide the reporting of this section.

### Aim

The aims of this study are to (a) identify the level of cultural influence on the RCM mechanisms and RFM items, and (b) provide recommendations to inform cross-cultural applicability of RCM and RFM.

### Study design and setting

This study follows Belton’s six-step Delphi methodology [23] to guide the process systematically and defensibly. Table 2 presents the application to our study. We will use the decentring approach to cross-cultural research. This approach aims to widen the use of tools developed in one cultural context to others, with careful attention to cultural relevance and potential revision as needed [20]. While van de Vijver and Leung originally used the term “the decentred approach”, [20] we adopt “the decentring approach” to emphasise our active engagement in analysing and adjusting tools across diverse cultural contexts.

**Table 2:** Application of Belton’s Six-Step Delphi Methodology.

Step		Aim	Activity	Outcome
1	Setting up a Delphi process	Recruit panellists	Invite RC experts via RIRC	Diverse international expert panel (n = 100)
2	Developing question items and response scales	Develop survey	Draft and pilot survey with five country leads; refine based on feedback	Finalised survey reflecting clarity and cross-cultural applicability
3	Software delivery choice	Conduct Delphi rounds	Collect quantitative (Likert, Type 1/2) and qualitative (open-ended) responses	Identified cultural challenges and proposed revision
4	Providing feedback to panellists	Provide feedback between rounds	Summarise medians, IQRs, key recommendations; solicit feedback	Refined RCM, RFM, and key recommendations
5	Preventing and dealing with panellist drop-out	Manage participation	Weekly reminders, optional support meetings, social recognition	Minimised attrition bias (>80% retention between rounds)
6	Analysing and presenting the Delphi data	Analyse and report findings	Non-parametric statistics; descriptive content analysis informed by Hofstede theory	Robust, culturally sensitive recommendations for RC enhancement

RC = Recovery College; RIRC = RECOLLECT International Research Consortium; IQR = Inter-Quartile Range; RCM = RECOLLECT Change Model; RFM = RECOLLECT Fidelity Measure.

The setting of the study is online: data collection will be conducted via online surveys, and data analysis and management will take place at King’s College London.

### Participants and recruitment

Participants, called “panellists” in the Delphi surveys, [24] will be recruited through the RECOLLECT International Research Consortium (RIRC) [25], which currently spans 31 countries across Africa, Asia, Europe, North America, South America, and Oceania (n = 36 as some positions are job-shared). Table 3 shows the 31 countries. RIRC country leads—who have been actively involved in RECOLLECT 2’s global studies, [2,11–14] and who bring both deep knowledge of RCs in their countries and diverse professional backgrounds—will be approached by the lead researcher who is the Delphi Administrator (YK).

**Table 3:** 31 countries in the RECOLLECT International Research Consortium.

Australia
Belgium
Brazil
Bulgaria
Canada
Czech Republic/ Czechia
Denmark
Estonia
Finland
France
Germany
Hong Kong
Hungary
Ireland
Italy
Japan
Netherlands
New Zealand/ Aotearoa
Norway
Poland
Portugal
Singapore
Spain
Sweden
Switzerland
Thailand
Uganda
England
Scotland
Wales
USA

### Alphabetical order

The Delphi Administrator will invite country leads to participate as panellists and to recruit additional eligible panellists. Eligibility criteria are: (a) a minimum of three years of experience with a RC in any role (staff, student, or researcher), and (b) 18 years or older. We aim to recruit approximately 100 panellists, exceeding recommended Delphi sample sizes [23]. This increases the likelihood of capturing a diverse range of cultural perspectives and enhances the reliability of subgroup comparisons across countries. Panellist consent will be implied by submission of responses, consistent with standard Delphi practice [26]. If responses remain low in some countries, the Delphi Administrator will follow up with country leads or relevant contacts to encourage participation. As soon as five responses are received from the same country, the Delphi Administrator will contact the panellists of that country, including the country leads, to request that recruitment be stopped in order to maintain balance and comparability across countries. Responses received after this point will still be included in the analysis, however we do not anticipate substantially more responses being submitted after such notifications. Recruitment will begin following protocol publication and will continue until seven consecutive days pass without receiving a new panellist response, to maximise participation while ensuring timely study progression.

The geographical dispersion of the panellists will be heavy in the Western countries, because there are many RCs in these countries (204 of 221 RCs in 2022) [13]. However, the numbers of RCs have been increasing in non-Western countries such as Japan since the time of the study in 2022 (11 RCs in 2022–20 in 2025; fourth largest number in the world). High engagement from non-Western countries will be feasible and strongly encouraged.

### Survey development

The survey was initially drafted by YK and reviewed by the research team. The initial draft was designed to address different cultural influences identified in our previous studies regarding the RC operational model [13,14]. It was piloted by five country leads (Brazil, Estonia, Germany, Japan, and Thailand) to ensure item clarity and relevance. Revisions were made based on pilot feedback (S1 File) to enhance cultural applicability and comprehension. Key changes resulting from the pilot included: (a) switching from Microsoft Word to Microsoft Forms for survey delivery, (b) adding clarifying examples to terms—for instance, examples of “RC non-peer trainer” such as psychologist, nurse, social worker, and educator were added, and (c) collapsing the five-point Likert response scale into three categories in the consensus analysis, and adding an optional comment box to capture nuance and address potential cross-cultural response bias (as detailed in the Data Analysis section below).

The finalised survey will be distributed to panellists. It includes questions asking panellists to rate or comment on:

1. The importance of each RCM mechanism and RFM non-modifiable item.
2. The cultural difficulty of meeting each RCM mechanism and RFM non-modifiable item.
3. Recommendations for culturally sensitive wording for each RCM mechanism and RFM non-modifiable item.
4. Which response type to RFM modifiable items aligns more with their country’s culture.

Quantitative questions use a five-point Likert scale; qualitative input will be gathered for recommendations.

The first two questions will be answered using a five-point Likert ordinal scale: 1 = “Not at all” to 5 = “Very important” for the first question, and 1 = “Not at all” to 5 = “Very difficult” for the second question. The third questions will be answered qualitatively to identify recommendations. The fourth questions will be responded either “Type 1” or “Type 2”. Please see S2 File for the finalised survey. Table 4 summarises the survey.

**Table 4:** Summary of the Delphi survey (Round 1).

No.	Question	Items	Response
**1**	Importance	RCM mechanisms and RFM non-modifiable items	Five-point Likert scale + optional comments
**2**	Cultural difficulty	RCM mechanisms and RFM non-modifiable items	Five-point Likert scale + optional comments
**3**	Wording recommendations	RCM mechanisms and RFM non-modifiable items	Open-text comments (optional)
**4**	Cultural alignment	RFM modifiable items	Type 1 or Type 2 selection + optional comments

### Data collection

Data will be collected via Microsoft Forms. Survey links will be emailed directly to panellists to ensure efficiency and accessibility [26] while maintaining quasi-anonymity (panellists do not know others’ identities, but researchers do) [27]. To maximise participation among panellists who are not fluent in English, support will be provided by country leads—all of whom are fluent in English—and the use of machine translation tools such as Google Translate, which are increasingly used in research [28], will be permitted. Formal translation procedures, such as forward and backward translation, will not be employed, as the maximum number of panellists per country is only five. The first round will remain open until seven consecutive days pass without a new response, to maximise participation while maintaining momentum. From the second round onwards, each round will be open for two weeks, followed by up to four weeks of analysis [29]. Three rounds are anticipated but flexibility is allowed based on consensus stability.

Continuous high engagement is essential for the success of Delphi studies [30]. To ensure continuous high engagement, weekly reminders will be used and an option to have an online meeting with the Delphi Administrator will be provided [31]. Financial incentives will not be used as they can damage the quality of the panellist input, for example by making panellists less conscientious or by making them feel their opinions are ‘bought’ by the research team [32]. Instead, social recognition will be offered to maximise meaningful continuous high engagement [23]. The panellists will be invited to be listed in the acknowledgements section of the paper [33].

### Data analysis

For the first two quantitative questions (importance and difficulty ratings), if at least 70% of responses select the same option, it will be regarded as consensus [34]. Diamond et al. (2014)’s systematic review reported consensus thresholds ranging from 50% to 97%, with a median of 75% [34]. Given the exploratory and cross-cultural nature of this study, we adopted a 70% threshold. To address potential cross-cultural response bias in this global study—such as extreme response styles or acquiescence [12]—we will collapse the original five-point Likert scale (“Not at all” = 1 to “Very important” or “Very difficult” = 5) into a three-point Likert scale. Responses 1 (“Not at all”) and 2 (“Not much”) will be merged into “Not important” or “Not difficult” (scored as 1), 3 (“Somewhat”) will be retained as “Somewhat” (scored as 2), and 4 (“Important” or “Difficult”) and 5 (“Very important” or “Very difficult”) will be merged into “Important” (scored as 3). This decision was informed by analysis of the five pilot responses, which indicated that a three-point scale would preserve meaningful distinctions while improving cross-cultural comparability. These pilot responses are provided in Table 5.

**Table 5:** Pilot responses for the importance and difficulty questions.

	How important each RCM and RFM non-modifiable item is to your RC (1 = Not at all; 5 = Very important)										
	RCM				RFM						
Pilot panellists	(a) empowering environment	(b) shifting the balance of power	(c) enabling different relationships	(d) facilitating personal growth	Equality	Learning	Tailor	Co-production	Social Connectedness	Community	Commitment to recovery
A	5	5	5	5	5	5	5	5	5	5	5
B	4	4	4	4	4	4	4	3	4	1	5
C	5	5	5	5	5	5	4	4	5	4	5
D	5	5	5	5	4	5	2	5	4	5	5
E	4	5	5	4	5	5	4	5	4	5	4
	**How culturally difficult it is to meet each RCM and RFM non-modifiable item for your RC (1 = Not at all; 5 = Very difficult)**										
	RCM				RFM						
**Pilot panellists**	**(a) empowering environment**	**(b) shifting the balance of power**	(c) enabling different relationships	**(d) facilitating personal growth**	**Equality**	**Learning**	**Tailor**	**Co-production**	**Social Connectedness**	**Community**	**Commitment to recovery**
A	2	4	2	3	4	3	3	3	1	2	1
B	4	4	3	2	2	3	2	2	3	4	1
C	3.5	3.5	2	3.5	1	2	2	5	2	3.5	1
D	1	3	3	3	2	1	Blank	2	1	2	1
E	3	2	2	4	2	4	2	2	3	1	3

> 70% consensus.

> 70% consensus if collapsed into 3-point Likert responses (1 and 2 as “Not important”, 3 as “Somewhat”, and 4 and 5 as “Important”).

Non-parametric statistical analyses will be conducted, including calculation of medians and interquartile ranges to summarise central tendency and dispersion. These are appropriate for ordinal data and will be used to examine patterns of agreement and stability across Delphi rounds. We will also explore changes in responses across rounds to assess the emergence of consensus. Any comments made in the optional comment box will be considered in the consensus interpretation.

For the fourth quantitative question (cultural alignment), the numbers and proportions of response types will be calculated. Additionally, all responses will be linked to Hofstede’s six cultural dimensions to explore whether patterns emerge between cultural orientation and item-level responses (e.g., countries with high Restraint tend to score high on a certain item). This approach allows us to move beyond broad regional classifications and potentially identify culturally sensitive insights that can inform adaptations for different cultural groups.

As recommended, [35,36] the quantitative results will be presented to the panellists after all rounds to prevent majority opinions causing opinion changes (e.g., less confident panellists may change their opinion to a majority opinion) [37].

The third section, comprising optional qualitative questions, will ask what changes are needed to the wording of each RCM mechanism and RFM non-modifiable item. The responses will be analysed using descriptive content analysis [38,39] to summarise panellists’ recommendations for improving the cultural relevance [40]. This approach is appropriate for Delphi studies where the aim is to capture clear, actionable feedback rather than develop theory or deep interpretation [41,42]. The analysis will follow the six analytic strategies [43,44]: (a) coding (b) recording insights and reflections, (c) identifying patterns and key features, (d) comparing similarities and differences, (e) generating descriptive generalisations, and (f) relating findings to existing literature for context. As with the quantitative data, the contextual interpretation of qualitative findings (step f) will draw on Hofstede’s cultural dimension theory to explore culturally grounded meanings and differences [21]. YK will conduct the initial grouping and summarisation, with review by co-authors. To enhance trustworthiness, finalised findings will undergo member checking by panellists to mitigate researcher bias [45,46].

In the second round, the panellists will be asked to assess whether the key recommendations would fit the cultural context of RCs in their country. If they assess a recommendation would not fit, they will be asked to suggest a revision. All suggested revisions will be analysed using descriptive content analysis, and integrated into RCM and RFM by YK, which will be reviewed by the rest of the co-authors. This process will generate (a) key recommendations to RC operation, RCM and RFM, and (b) revised RCM and RFM. These will be presented to the panellists.

These steps will be repeated until the panellists agree with the key recommendations. In Delphi literature, three rounds are generally considered enough, [47]. however we will consider the stability of consensus or dissensus as the criterion to finish the Delphi consultation [23].

Visual aids will be used where possible to present our findings, because the audience of our Delphi research will be wide including RC staff, students and researchers [23]. The language used will also be inclusive of the wide audience, which will be informed by the panellists with diverse cultural and professional backgrounds as well as RC experiences.

### Data management plan

All data will be securely stored on King’s College London servers accessible only to the research team. Participant confidentiality will be maintained according to the General Data Protection Regulation standards. After study completion, fully anonymised data will be made publicly available via the Open Science Framework to support transparency and reproducibility. Anonymisation will follow a strict protocol to remove any direct or indirect identifiers. Where necessary, further de-identification techniques such as data aggregation or suppression will be applied to ensure no individual can be re-identified, in line with best practices for high-level anonymisation [48,49]. Data aggregation involves grouping specific details into broader categories (e.g., reporting “a city in Japan” instead of “Kyoto”), while data suppression refers to omitting uniquely identifying details altogether (e.g., replacing a country name with “a European country” when fewer than four RCs exist there). These techniques protect confidentiality while preserving the value of the dataset. We will adopt a balanced model of anonymisation, aiming to maximise the protection of participants’ identities while maintaining the value and integrity of the data [49,50].

### Safety considerations

No safety risks are anticipated in this study.

### Ethical considerations

Approval was obtained from King’s College London Research Ethics Psychiatry Nursing and Midwifery Subcommittee (Reference: MRM-24/25–47085). Participation will be voluntary with informed consent required.

### Study status and timeline

The pilot phase has been completed. Main data collection will commence following publication of the protocol, with study completion expected within 12 months.

## Discussion

This study will identify RCM and RFM components that present cultural challenges and those that are widely acceptable across different countries. These insights will highlight areas requiring refinement and inform strategies to enhance cultural inclusivity in RC operations, the RCM, and the RFM. Through the iterative Delphi process, consensus-based recommendations will be developed to support the culturally responsive implementation of RCs, particularly in regions where cultural considerations have been under-recognised. These findings will contribute to the global expansion of RCs while ensuring their relevance across diverse contexts.

This Delphi study will advance the understanding of cultural influences on RC mechanisms and operational components, [14] aiming to enhance their global relevance and effectiveness. We expect to identify which elements of the RCM and RFM are widely applicable, which may require refinement to support broader cross-cultural applicability. In doing so, the study will contribute to addressing the widely recognised tension between fidelity and fit in cross-cultural research [51,52]. By identifying both widely applicable and culturally specific elements of RC practices, the study will help clarify how core components can be maintained (ensuring fidelity) while allowing appropriate modification to improve relevance and acceptability across diverse contexts (ensuring fit). These insights are expected to benefit researchers seeking to implement evidence-based tools globally, as well as staff and students aiming to strengthen RCs in their own cultural settings.

Achieving a stable consensus is often regarded as a central aim in Delphi studies. However, in the context of a highly diverse international panel, dissensus—persistent disagreement—can be equally informative [53]. Therefore, both consensus and dissensus will be valued outcomes. Areas of consensus will provide a foundation for core RC practices, while areas of dissensus will highlight culturally specific considerations, informing context-sensitive refinements [52]. These insights will not only be useful at the national level, but also at regional or organisational levels. For example, ethnic minority communities whose cultural values differ from dominant national norms may feel more included by recognising culturally diverse input.

The inclusion of approximately 100 panellists from more than 30 countries will significantly strengthen the credibility and global applicability of the findings. The use of the RIRC network ensures that participants will have deep knowledge of RC operations in varied cultural contexts, and the pilot testing process ensures that survey items are accessible and meaningful across different countries. This high level of diversity will help mitigate the over-representation of Western perspectives that has limited the evidence base for RCs [11].

Our methodological approach draws on recent best practices for Delphi studies (e.g., [23,24,33]), including quasi-anonymity, controlled feedback, and assessment of response stability across rounds. While large-scale cross-cultural Delphi studies in mental health are relatively rare, this study adds methodological strength through its combination of statistical and theory-informed qualitative analysis. By adopting a member-checking process after the final round, we aim to ensure that the findings are trustworthy and reflective of panellists’ views [45,46]. The integration of non-parametric statistical analysis and descriptive content analysis informed by Hofstede’s cultural dimensions [21] provides a robust mixed-methods framework for identifying culturally relevant insights.

Anticipated outcomes include a set of practical recommendations for refining the RCM and RFM to enhance their cross-cultural applicability (e.g., strategies to address translational gaps or culturally sensitive terminology). These recommendations are expected to enhance the inclusivity and effectiveness of RCs internationally, particularly in non-Western settings where cultural differences may otherwise pose barriers to successful implementation.[20]. Moreover, enhancement to the RCM and RFM will be proposed, offering updated frameworks that better accommodate global variations in recovery practices.

### Limitations

Limitations of the study include the possibility of uneven geographical representation, particularly if panellist recruitment remains skewed toward Western countries where RCs are more established. Efforts will be made to mitigate this by encouraging high engagement from non-Western countries. Using Hofstede’s metrics, we will compare data at the country level. However, we acknowledge that different cultural orientations may exist within a country such as regional, occupational, or generational cultures, which will not be assessed in depth in this study. Additionally, conducting the study entirely in English may pose accessibility challenges for some participants, especially where the linguistic distance—how structurally and lexically different two languages are [54]—from English is substantial. To address this, translation support will be offered by country leads (fluent in English), and machine translation will be allowed where needed. Future research could explore multilingual approaches to broaden participation even further.

### Dissemination

Dissemination of findings will occur through publication in peer-reviewed journals, presentations at international mental health conferences, and public-facing events and materials (e.g., recovery-related websites such as “Research into Recovery” https://www.researchintorecovery.com/). We anticipate that these outputs will support policymakers, practitioners, and researchers in tailoring RC models to better serve culturally diverse populations.

### Amendments

We do not anticipate any amendments to the study protocol. However, potential areas where changes may arise include the timeframes for panellist recruitment and data analysis, depending on participation rates and data volume. Should any amendments become necessary, they will be clearly documented and reported in the final study manuscript. In the unlikely event of study termination, all panellists will be informed directly by the research team.

## Conclusion

This study aims to strengthen and expand the global relevance of RCs by promoting cross-cultural applicability and inclusion in mental health recovery. By identifying both shared principles and culturally specific considerations, the study will contribute to more inclusive and responsive RC frameworks. As the need for RCs grows worldwide, we hope this research will support their meaningful expansion across diverse settings. Ultimately, we aim for people engaged with RCs around the world to feel that their cultural perspectives are recognised and reflected in RC practices and tools, helping improve services and research, and bring greater benefit to the communities they serve.

## Supporting information

S1 File Pilot feedback on survey. (DOCX)

S2 File Delphi Round 1: Enhancing cultural adaptability in Recovery Colleges. (DOCX)
