# Supplementary material for: Enhancing cross-cultural applicability in recovery colleges: A global Delphi study protocol
Source: PLoS One. 2025 Sep 30;20(9):e0332729. doi: 10.1371/journal.pone.0332729 (PMC12483229; doi:10.1371/journal.pone.0332729)
Supplement: S1 File — (DOCX) [file pone.0332729.s001.docx]

# S1 Supporting Information

# Pilot feedback on survey

| **About Your RC** | **Importance** | **Cultural Difficulty** | **Wording Recommendation** | **Cultural Alignment** | **Other Comments** |
| --- | --- | --- | --- | --- | --- |
| "RC non-peer trainer" - What is the difference between a non-peer trainer and other RC staff? This is where confusion can arise. For example, under which category would a psychologist or social worker who is one of the trainers fall? And could there be more than one answer?  "Which city and country, is your RC(s) based?" - If there are e.g. 2 RCs in one city, is there any way to distinguish them here? If an organisation is active in more than one city, how should it be indicated here?  If I understand correctly, you are planning to use this word document for data collection? It might be worthwhile to dedicate a few hours to implement the questionnaire in some sort of online survey tool, it will save time in the end. |  |  | [About the layout of the choices] Maybe consider using bullet points. It is difficult to read all this text. |  | Completing the survey took around 20 minutes. I think this is a very acceptable time.   The end of the survey is a bit abrupt. It would be nice to have some sort of thank you for your time. Would you be ok to be contacted again for the next round. Here is an overview of the next steps. Here is what we plan to do next. etc  We are still implementing an RC in [place]. We are planning to begin activities in [time]. |
