# Supplementary material for: Enhancing cross-cultural applicability in recovery colleges: A global Delphi study protocol
Source: PLoS One. 2025 Sep 30;20(9):e0332729. doi: 10.1371/journal.pone.0332729 (PMC12483229; doi:10.1371/journal.pone.0332729)
Supplement: S2 File — (DOCX) [file pone.0332729.s002.docx]

# S2 Supporting Information

# Delphi Round 1: Enhancing cultural adaptability in Recovery Colleges

Thank you for participating as a panellist in this study. Recovery Colleges (RCs) are being used globally, and we are finding that some aspects of RCs are influenced by cultures. So, we would like to improve the cultural adaptability of RC operation and tools. To understand *how* to improve cultural adaptability, we would like you to respond to some questions about the RECOLLECT Change Model (RCM) and RECOLLECT Fidelity Measure (RFM) over 2-3 rounds. Approval was obtained from King’s College London Research Ethics Psychiatry Nursing and Midwifery Subcommittee on 10/02/25 (MRM-24/25-47085).

**RECOLLECT Change Model (RCM)**

The RCM explains how RCs work. The key mechanisms are: (a) empowering environment; (b) shifting the balance of power; (c) enabling different relationships; and (d) facilitating personal growth (you can access the figure [here](https://bmcpsychiatry.biomedcentral.com/articles/10.1186/s12888-022-04253-y/figures/1)).

**RECOLLECT Fidelity Measure (RFM)**

RFM evaluates RC adherence to core operational components. There are 12 components, comprising seven non-modifiable components (Equality, Adult Learning, Tailoring to the Student, Co-production, Social Connectedness, Community Focus and Commitment to Recovery) and five modifiable components (Available to All, Location, Distinctiveness of Course Content, Strengths-based, and Progressive). Each component is assessed by the RC manager rating one item.

In Round 1, please answer questions about your RC profile and culture.

## Q1. What is your primary role in your RC? (select one)

RC manager

RC peer trainer

RC non-peer trainer (e.g., psychologist, nurse, social worker, educator)

Other RC staff (please specify): _______________

RC researcher

Other (please specify): _____________

## Q2. How long have you been involved with this or any other RC? (select one)

Less than 1 year

1–3 years

4–6 years

7+ years

Q3. In which city and country is your RC (or RCs) based? If you are involved in multiple RCs, please list the cities and countries in order of your involvement, starting with the one you are most involved in.

__ ___________________________________

Q4. What is the main organisational affiliation for your RC (e.g. whose buildings you operate from or where data and records is stored (choose one, or more than one if an equal partnership))?

Government health service

Other health provider, e.g. private healthcare provider

Local Government

Education provider, e.g. University or college

Non-governmental organisation (NGO) or Charity Sector

We are independent

Other (please specify): _____________

## Q5. Please enter your email address (to be used next rounds)

___________________________________________________

## Q6. We would like to acknowledge you in the Acknowledge section of a research paper from this study. If that is okay with you, please enter your name.

___________________________________________________

## Q7. Please indicate **how important** each RCM mechanism and RFM non-modifiable item is to your RC.

| **RCM** | **How important** |
| --- | --- |
| (a) empowering environment | Not at all – Not much – Somewhat – Important – Very important |
| (b) shifting the balance of power | Not at all – Not much – Somewhat – Important – Very important |
| (c) enabling different relationships | Not at all – Not much – Somewhat – Important – Very important |
| (d) facilitating personal growth | Not at all – Not much – Somewhat – Important – Very important |
| **Optional: Any comments (e.g., why you think certain items are important/unimportant to your RC)** |  |

| **RFM non-modifiable items** | **How important** |
| --- | --- |
| **1. Valuing equality**  The contributions and assets of students, trainers (peers, clinicians, external) and other staff are equally valued. No one is judged or treated differently because of their background or mental health difficulties. | Not at all – Not much – Somewhat – Important – Very important |
| **2. Learning**  Recovery Colleges follow an adult education approach whereby students and trainers collaborate and learn from each other by sharing experiences, knowledge and skills. Students have responsibility for their learning and learn through interactive and reflective exercises. Students gain self-awareness, understanding of their difficulties and practical, relevant self-management skills. Students choose courses which best suit their interests and aspirations. | Not at all – Not much – Somewhat – Important – Very important |
| **3. Tailored to the student**  Recovery Colleges don’t offer a one-size-fits-all experience. Students’ individual needs are actively enquired about and accommodated during courses (e.g. personalised handouts, translated text, materials adapted for learning difficulties). Their needs outside the course are also accommodated (e.g. buddy service, transport help, individual learning plans). | Not at all – Not much – Somewhat – Important – Very important |
| **4. Co-production of the Recovery**  **College**  People with lived experience (Peer  Trainers and students) are brought together with professionals and subject experts to design and deliver all aspects of the Recovery College. This includes collaborative decision making about the prospectus, courses, college policies, staff recruitment, advertising, etc., as well as the co-design and co-delivery of all courses by a Peer Trainer and other subject-expert. | Not at all – Not much – Somewhat – Important – Very important |
| **5. Social connectedness**  Both the culture and the physical environment of the college provide students with opportunities to develop connections with others. The learning space is relaxed, e.g. nonclinical chair layout, access to drinks facilities, shared spaces for socialising. Trainers recognise and  cater for students' social needs, e.g. organising exercises and breaks for chatting, sharing experiences and  developing friendships. | Not at all – Not much – Somewhat – Important – Very important |
| **6. Community focus**  Recovery Colleges engage with community organisations (e.g. mental health charities, artistic/sporting groups) and Further Education colleges to co-produce relevant courses. The college provides students with information, handouts and events which support students' pathways into valued activities, roles, relationships and support in the community. | Not at all – Not much – Somewhat – Important – Very important |
| **7. Commitment to recovery**  Recovery College workers talk with conviction and enthusiasm about the service and are dedicated to students' recovery. There is a positive energy in the college and its activities, based on shared values about the recovery principles on which the college is based. | Not at all – Not much – Somewhat – Important – Very important |
| **Optional: Any comments (e.g., why you think certain items are important/unimportant to your RC)** |  |

## Q8. Please indicate **how culturally difficult it is to meet** each RCM mechanism and RFM non-modifiable item for your RC.

| **RCM** | **How culturally difficult** |
| --- | --- |
| (a) empowering environment | Not at all – Not much – Somewhat – Difficult – Very difficult |
| (b) shifting the balance of power | Not at all – Not much – Somewhat – Difficult – Very difficult |
| (c) enabling different relationships | Not at all – Not much – Somewhat – Difficult – Very difficult |
| (d) facilitating personal growth | Not at all – Not much – Somewhat – Difficult – Very difficult |
| **Optional: Any comments (e.g., why you think certain items are culturally difficult/easy; if difficult, what you did to meet them)** |  |

| **RFM non-modifiable items** | **How culturally difficult** |
| --- | --- |
| **1. Valuing equality**  The contributions and assets of students, trainers (peers, clinicians, external) and other staff are equally valued. No one is judged or treated differently because of their background or mental health difficulties. | Not at all – Not much – Somewhat – Difficult – Very difficult |
| **2. Learning**  Recovery Colleges follow an adult education approach whereby students and trainers collaborate and learn from each other by sharing experiences, knowledge and skills. Students have responsibility for their learning and learn through interactive and reflective exercises. Students gain self-awareness, understanding of their difficulties and practical, relevant self-management skills. Students choose courses which best suit their interests and aspirations. | Not at all – Not much – Somewhat – Difficult – Very difficult |
| **3. Tailored to the student**  Recovery Colleges don’t offer a one-size-fits-all experience. Students’ individual needs are actively enquired about and accommodated during courses (e.g. personalised handouts, translated text, materials adapted for learning difficulties). Their needs outside the course are also accommodated (e.g. buddy service, transport help, individual learning plans). | Not at all – Not much – Somewhat – Difficult – Very difficult |
| **4. Co-production of the Recovery**  **College**  People with lived experience (Peer Trainers and students) are brought together with professionals and subject experts to design and deliver all aspects of the Recovery College. This includes collaborative decision making about the prospectus, courses, college policies, staff recruitment, advertising, etc., as well as the co-design and co-delivery of all courses by a Peer Trainer and other subject-expert. | Not at all – Not much – Somewhat – Difficult – Very difficult |
| **5. Social connectedness**  Both the culture and the physical environment of the college provide students with opportunities to develop connections with others. The learning space is relaxed, e.g. nonclinical chair layout, access to drinks facilities, shared spaces for  socialising. Trainers recognise and cater for students' social needs, e.g. organising exercises and breaks for chatting, sharing experiences and developing friendships. | Not at all – Not much – Somewhat – Difficult – Very difficult |
| **6. Community focus**  Recovery Colleges engage with community organisations (e.g. mental health charities, artistic/sporting groups) and Further Education colleges to co-produce relevant courses. The college provides students with information, handouts and events which support students' pathways into valued activities, roles, relationships and support in the community. | Not at all – Not much – Somewhat – Difficult – Very difficult |
| **7. Commitment to recovery**  Recovery College workers talk with conviction and enthusiasm about the service and are dedicated to students' recovery. There is a positive energy in the college and its activities, based on shared values about the recovery principles on which the college is based. | Not at all – Not much – Somewhat – Difficult – Very difficult |
| **Optional: Any comments (e.g., why you think certain items are culturally difficult/easy; if difficult, what you did to meet them)** |  |

## Q9. What **changes** need to be made **to the wording of the RCM mechanisms and RFM non-modifiable items**, so that these mechanisms and items are more in line with the culture in your country? Please skip items that do not require changes.

| **RCM** | **Changes to Item** |
| --- | --- |
| (a) empowering environment |  |
| (b) shifting the balance of power |  |
| (c) enabling different relationships |  |
| (d) facilitating personal growth |  |

| **DIMENSION** | **CURRENT STAGE OF DEVELOPMENT** |
| --- | --- |
| **1. Valuing equality**  The contributions and assets of students, trainers (peers, clinicians, external) and other staff are equally valued. No one is judged or treated differently because of their background or mental health difficulties. | **Response for Score “0”**  We recognise that staff and students may take time to develop partnership-based working relationships. Whilst being supportive of staff and students, we only deal with issues of discrimination and power differences when they arise. |
|  | **Response for Score “1”**  We do not actively ensure that all relationships in the college demonstrate equal sharing of opportunities, training, etc. However, we do ensure that the college is welcoming to all staff and students, and have some structures in place (e.g. open days, training, supervision) to encourage equality and to challenge stigma and discrimination. |
|  | **Response for Score “2”**  We actively promote a non-judgemental and welcoming culture. Activities are undertaken to ensure that issues of power are always considered within the college (e.g. equal access to training and resources, diversity in promotional materials, analysing equal opportunity data). |
| **Changes to Item** |  |

| **DIMENSION** | **CURRENT STAGE OF DEVELOPMENT** |
| --- | --- |
| **2. Learning**  Recovery Colleges follow an adult education approach whereby students and trainer collaborate and learn from each other by sharing experiences, knowledge and skills.  Students have responsibility for their learning and learn through interactive and reflective exercises. Students gain self-awareness, understanding of their difficulties and practical, relevant self-management skills.  Students choose courses which best suit their interests and aspirations. | **Response for Score “0”**  We cannot provide evidence of the college’s model(s) of adult learning. We can identify a large number of barriers to progress, such as the influence of a strong clinical or psycho- educational model, or limited resources for Peer Trainer training. Trainers are skilled in delivering education and encouraging shared learning. |
|  | **Response for Score “1”**  We can articulate the college’s model(s) of adult learning. Some processes are in place to ensure that trainers follow educational principles (e.g. lesson plans, educational language) and that courses involve co-learning. However, some barriers prevent the full and effective implementation of these model(s), e.g. time pressures to launch/recruit to new courses, or barriers to trainer recruitment and training. |
|  | **Response for Score “2”**  We can demonstrate the college’s full commitment to principles of adult learning. These are evident in the college’s prospectus, curriculum and course materials. All trainers (including clinical trainers) can describe the model(s) of adult learning used in the college, and are offered ongoing formal or accredited training in adult learning. |
| **Changes to Item** |  |

| **DIMENSION** | **CURRENT STAGE OF DEVELOPMENT** |
| --- | --- |
| **3. Tailored to the student**  Recovery Colleges don’t offer a one- size-fits-all experience. Students’ individual needs are actively enquired about and accommodated during courses (e.g. personalised handouts, translated text, materials adapted for learning difficulties). Their needs outside the course are also accommodated (e.g. buddy service, transport help, individual learning plans). | **Response for Score “0”**  We are not able to demonstrate the ways in which the college provides an individualised experience for students. Trainers are not actively supported or trained to take account of and accommodate student differences during classes. |
|  | **Response for Score “1”**  We can demonstrate some ways in which individual needs of students are addressed, but recognise that there are still unmet needs, e.g. students with learning difficulties or difficulty expressing themselves. |
|  | **Response for Score “2”**  We are able to demonstrate many ways in which students’ individual needs are addressed both during and outside courses. Trainers are made aware of students' needs in advance and provided with guidance on how to adapt the content/delivery of courses. |
| **Changes to Item** |  |

| **DIMENSION** | **CURRENT STAGE OF DEVELOPMENT** |
| --- | --- |
| **4. Co-production of the Recovery College**  People with lived experience (Peer Trainers and students) are brought　 together with professionals and subject experts to design and deliver all aspects of the Recovery College. This includes collaborative decision- making about the prospectus, courses, college policies, staff recruitment, advertising, etc., as well as the co-design and co-delivery of all courses by a Peer Trainer and other subject-expert. | **Response for Score “0”**  We routinely involve students and staff in decision-making about the design and running of the Recovery College. Most of our success in co-production has been at the level of course co-delivery. We recognise that there are currently some significant barriers to co-production throughout the college, including those of culture, management hierarchies and time. |
|  | **Response for Score “1”**  As well as consistent co-delivery of courses, we involve staff and students in most discussions about the design and running of the Recovery College (e.g. through student steering groups or student reps), but managers make many of the decisions. |
|  | **Response for Score “2”**  We can demonstrate a culture of co-production and its consistent use across the college. The voices of trainers and students are equally heard during decision-making across all levels of the college, including co-delivery, curriculum development, management and design of the physical environment. |
| **Changes to Item** |  |

| **DIMENSION** | **CURRENT STAGE OF DEVELOPMENT** |
| --- | --- |
| **5. Social connectedness**  Both the culture and the physical environment of the college provide students with opportunities to develop connections with others. The learning space is relaxed, e.g. non- clinical chair layout, access to drinks facilities, shared spaces for socialising. Trainers recognise and cater for students' social needs, e.g. organising exercises and breaks for chatting, sharing experiences and developing friendships. | **Response for Score “0”**  Students' social experience is low on the Recovery College's agenda when deciding on course structure and the physical environment. There are no specific processes for students to get to know one another. Course venues rarely have facilities or spaces outside the classroom where students can relax or socialise. |
|  | **Response for Score “1”**  We ensure that the Recovery College is a welcoming environment for students. Trainers are encouraged to provide opportunities for socialising during courses where possible, but this is not central to their role. A few of our course spaces have facilities outside the classroom where students can relax, but there are a number of practical or financial barriers to this. |
|  | **Response for Score “2”**  The Recovery College recognises the role that student integration and connectedness plays in learning and recovery. The college provides a range of facilities for socialising (e.g. café, seating areas, informal and spacious course venues). Trainers are supported to integrate opportunities for students to form closer bonds with each other into the structure of courses. |
| **Changes to Item** |  |

| **DIMENSION** | **CURRENT STAGE OF DEVELOPMENT** |
| --- | --- |
| **6. Community focus**  Recovery Colleges engage with community organisations (e.g. mental health charities, artistic/sporting groups) and Further Education colleges to co-produce relevant courses. The college provides students with information, handouts and events which support students' pathways into valued activities, roles, relationships and support in the community. | **Response for Score “0”**  We have limited involvement with, or presence in, community organisations. Community organisations are not involved in college meetings or events, or do not routinely work with the college to co-produce courses or facilitate opportunities for staff/students. |
|  | **Response for Score “1”**  We ensure that the college undertakes some activities to build awareness of its community services and relationships with community organisations. Some college courses are co- produced with community organisations and students are signposted to relevant community organisations for support. |
|  | **Response for Score “2”**  We work with a range of community organisations to co-produce college courses and facilitate pathways for students. We can demonstrate activities to build awareness of, and relationships with, the community. We can demonstrate that joint-working with community organisations has led to changes in the college. |
| **Changes to Item** |  |
|  |  |
| **DIMENSION** | **CURRENT STAGE OF DEVELOPMENT** |
| **7. Commitment to recovery**  Recovery College workers talk with conviction and enthusiasm about the service and are dedicated to students' recovery. There is a positive energy in the college and its activities, based on shared values about the recovery principles on which the college is based. | **Response for Score “0”**  Our organisational policies and procedures ensure the Recovery College runs smoothly, but there are barriers (e.g. culture, organisational structures) to personal investment by workers in promoting recovery principles (dimensions 1 to 6 above) throughout the college. There is still significant effort needed to establish the college as something ‘different’ and ‘meaningful’. |
|  | **Response for Score “1”**  We actively motivate each other to promote recovery principles. We have a shared commitment to constantly improve the recovery focus of the college but recognise some barriers to progress (e.g. cultural, financial). |
|  | **Response for Score “2”**  We actively promote recovery principles in the college, and collectively lead with enthusiasm and an expressed belief in the college’s students and staff. College activities demonstrate recovery principles in practice, e.g. graduation ceremonies, students becoming trainers. |
| **Changes to Item** |  |

## Q10. Please indicate **which response type** to RFM modifiable items is more aligned with the culture of the country where your RC is located.

| **RFM modifiable item** | **Two response types** |
| --- | --- |
| **8. Available to all**  Recovery Colleges vary in the ways in which they implement eligibility criteria for student access. | **TYPE 1**  **The Recovery College is available to all.**  The Recovery College is accessible to any adult (16+ or 18+), including staff and carers, regardless of their use of local services of any kind. Any restrictions are minimal, e.g. living locally, being registered with a GP.  **TYPE 2**  **The Recovery College is limited to specific groups.**  The Recovery College is open to adults (16+ or 18+) who are current or previous users of local secondary care mental health services. There may be local additions to this eligibility e.g. health/social care/community organisation staff, or family and carers. Being ‘inclusive’ relates to the ways in which the Recovery College does not discriminate or create access barriers for people with, for example, certain diagnoses, learning difficulties or physical health/mobility needs. |
| **Optional: Any comments (e.g., why you chose that type)** |  |

| **RFM modifiable item** | **Two response types** |
| --- | --- |
| **9. Location**  Recovery Colleges vary in where courses are run. | **TYPE 1**  **The Recovery College is based in a community location that is not shared with health, social care or other statutory services.**  The Recovery College is deliberately located within communities or neighbourhoods, not in health or social care buildings.  **TYPE 2**  **The Recovery College is based in a location which is shared with health, social care or other statutory**  **services.**  The Recovery College is located within or near (e.g. adjoining building) to local health or social care services. |
| **Optional: Any comments (e.g., why you chose that type)** |  |

| **RFM modifiable item** | **Two response types** |
| --- | --- |
| **10. Distinctiveness of**  **course content**  Recovery Colleges vary in the content/subject matter of courses offered. | **TYPE 1**  **Any topic can be offered as a course, irrespective of whether it is available in mainstream adult education settings.**  The curriculum includes courses on topics which are also available in local mainstream colleges. Example courses might include gardening, arts, Maths, English, budgeting, understanding benefits, physical health care, job-seeking, home maintenance and a range of leisure/recreation activities.  **TYPE 2**  **Only topics not available in mainstream adult education settings are offered.**  The curriculum never includes courses on topics which are available in local mainstream colleges. Some courses are offered with a specific recovery-related focus, e.g. gardening for wellbeing, arts for recovery. |
| **Optional: Any comments (e.g., why you chose that type)** |  |

| **RFM modifiable item** | **Two response types** |
| --- | --- |
| **11. Strengths-based**  A strengths-based approach (focussing on assets and potential, not on problems) is either explicit or implicit within the language, courses and materials of the Recovery College. | **TYPE 1**  **A focus on strengths (not problems) is implicit in the college.**  The learning opportunities offered by the Recovery College implicitly builds on the experiences, strengths, assets and resources of students. The language of being ‘strengths-based’ is not often used.  **TYPE 2**  **A focus on strengths (not problems) is explicit in the college, in addition to dimensions 1-7 above (non-modifiable items).**  The learning opportunities offered by the Recovery College explicitly build on the experiences, strengths, assets and resources of students. The language of being ‘strengths-based’ is routinely used by staff and students, and features in course materials and other aspects of the Recovery College. |
| **Optional: Any comments (e.g., why you chose that type)** |  |

| **RFM modifiable item** | **Two response types** |
| --- | --- |
| **12. Progressive**  There is variation in the ways in which Recovery Colleges focus on, enable and encourage the forward-moving, goal-focused nature of the student experience. | **TYPE 1**  **There is a focus on ‘being’ and ‘belonging’, not on goal-setting.**  The focus of the Recovery College is on supporting individual students' learning needs, safety and belonging, identity development, personal meaning-making and reflection. The college does not require behavioural goal-setting. Students can learn in whatever direction they want to – and for some students that might not be about moving forwards.  **TYPE 2**  **There is a focus on ‘becoming’ and a strong emphasis on goal-setting and change.**  The focus of the Recovery College is on processes which provide pathways of opportunity for students and which support them to move on with their lives. This might include the use of goal-oriented personal plans (Individual Learning Plans) and planning and reviewing goal-oriented activities. |
| **Optional: Any comments (e.g., why you chose that type)** |  |

**Any comments in general (optional):**

|  |
| --- |

Thank you very much for your valuable input. We will carefully integrate your feedback and share the next steps with you soon.
